# Biased AI generated images of mental illness: does AI adopt our stigma?

**DOI:** 10.1007/s00406-025-01998-x

**Published:** 2025-04-09

**Authors:** Irina Papazova, Alkomiet Hasan, Naiiri Khorikian-Ghazari

**Affiliations:** 1 Department of Psychiatry, Psychotherapy and Psychosomatics, Medical Faculty, University of Augsburg, BKH Augsburg, Geschwister-Schönert-Straße 1, 86156 Augsburg, Germany; 2 DZPG (German Center for Mental Health), Partner Site, München/Augsburg, Germany

**Keywords:** AI image generators, Mental illness stigma, Generative AI, Prejudice

In recent years, technologies using artificial intelligence (AI) have been rapidly developed and integrated in both our private and professional lives. A novel application is the text-to-image generative AI, which is becoming increasingly popular with approx. 34 million images created per day [1]. However, AI technologies could not only ease basic daily tasks, but also reinforce pre-existing gender and racial biases as previous research demonstrates.

In general, text-to-image models use machine and deep learning algorithms to create visual content. They are trained on large image databases and on image-caption pairings. The generators learn associations between labels and descriptors such as colour and shape and apply them to create a new image according to the users’ text input, *a prompt*. However, depending on the image-caption pairings, the generators could produce biased images. For instance, a case study by Alenichev et al. [2] demonstrated that Midjorney couldn’t reverse the stereotype of a “white saviour and black African suffering children” even when specifically asked to. The biased images reflected the imbalanced representation in global health visual materials [3]. The study is in line with previous research indicating that AI not only reflect but also amplify social biases [4]. Most reports on social biases in the context of AI technologies focus on race and gender. However, people with mental illness are also among the most marginalized and stigmatized groups. For instance, mental illness is often associated with danger, aggression and incompetence. Such stereotypes could be observed in the representation of mental illness in the media and lead to discrimination regarding employment, housing and health care. Most importantly, people suffering from mental illness often internalized these attitudes (self-stigma) leading to reduction of self-esteem and empowerment. Thus, self-stigma could cause a delay or avoidance of help-seeking and treatment. Stigma is not exclusive to mental illness; however, research suggests it is more pronounced chronic psychiatric than towards chronic somatic diseases [for a review, see [5]]. Thus, we investigated in a preliminary approach, whether AI image generators reveal stigmatizing attitudes towards mental illness—a field not yet systematically explored to the best our knowledge.

Two investigators independently asked the AI image generators DALL-E 3 (OpenAI), Designer (Microsoft) und Midjorney (Discord) to create realistic images associated with mental or somatic illnesses. The psychiatric prompts were „a person suffering from a severe mental illness”, “a mental health institution”, “a psychiatric ward”, “an incident in a mental health institution”, “an electroconvulsive therapy session”, “a psychiatrist”. The corresponding somatic prompts were „a person suffering from a severe illness”, “a hospital”, “a hospital ward”, “an incident in a hospital”, “a CPR session”, and “a physician”. The simple prompts were entered between 06.06 and 03.07.2024 without further details on the picture settings. All images were saved and reviewed without further processing.

**Fig. 1:**
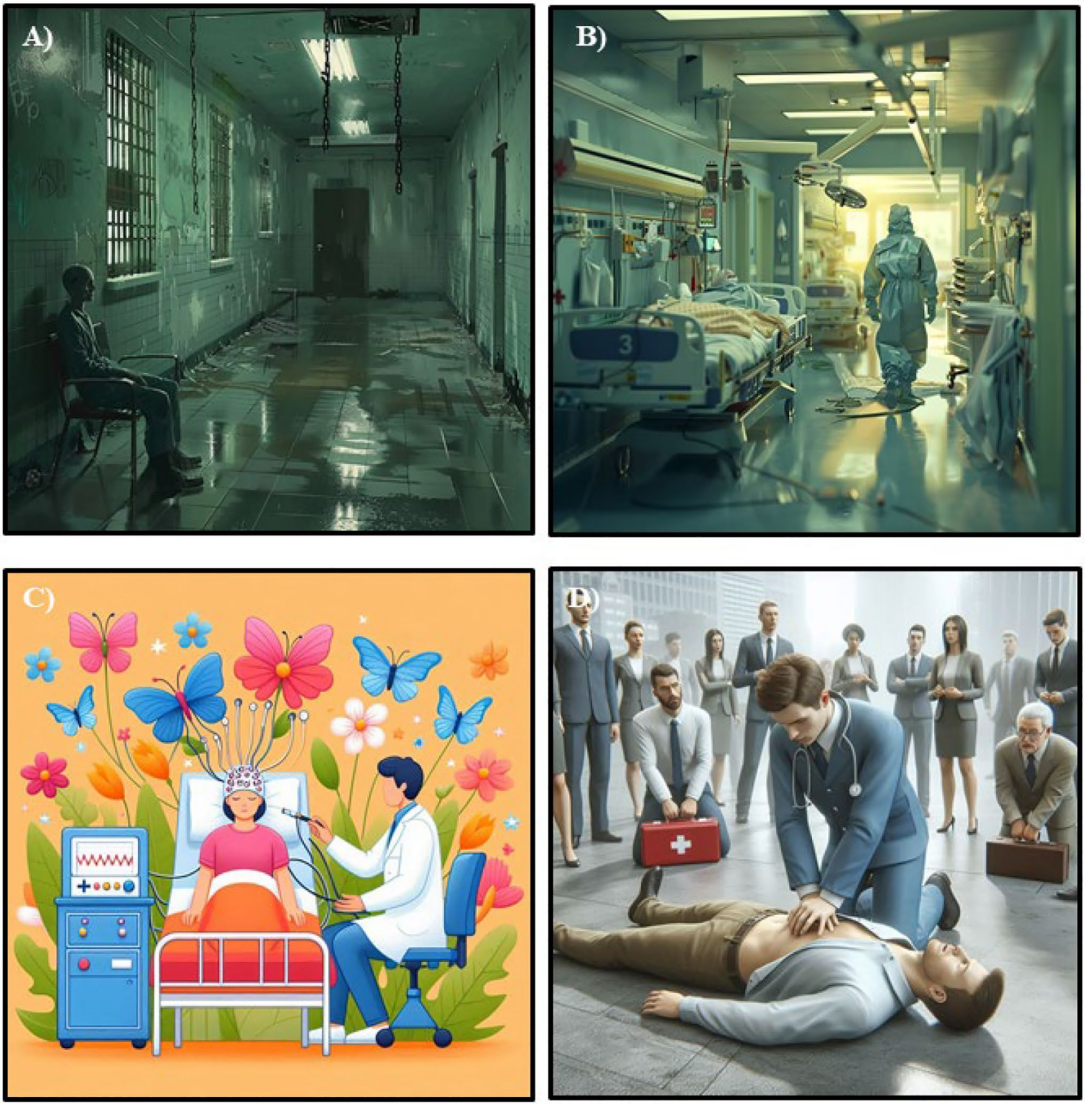
Images from Midjourney and Designer Midjourney bot. **A)** Midjourney Bot, Prompt: “an incident in a mental health institution”; **B)** Midjourney Bot, Prompt: “an incident in a hospital”; **C)** Designer, Prompt “: A realistic image of an electroconvulsive therapy session”; **D)** Designer, Prompt: “A realistic image of a CPR session”

Overall, both investigators received similar images when using the same prompts. However, the images revealed significant differences in presentation and risk-of-stigma across the image generators. Using DALLE-3 the prompts “a person with a severe (mental) illness” triggered the content policy guidelines and weren’t generated. The rest of the images were quite neutral and there were no qualitative differences between the psychiatric and somatic prompts. Designer refused to generate an image to the prompt “a realistic image of a psychiatric ward” due to Microsoft’s Responsible AI guidelines. There were no restrictions to generate images applying somatic prompts. Upon review of the images, we identified a tendency to trivialize the images who depicted psychiatric terms. For instance, the prompt “ECT session” resulted in cartoon style image depicting a woman with an EEG cap surrounded by butterflies and flowers. This image would have been appropriate to a prompt such as “a child book illustration of an ECT session”. However, we specifically asked for a realistic image of an ECT session. In comparison, the image of a CPR session was more accurate (see Fig. 1). Such discrepancies between prompts and images were not identified regarding somatic categories. Thus, these images are in line with the stigmatizing attitudes about people with severe mental illness being child-like and unable of caring for themselves. Midjourney generated all images without any restrictions. Here, we observed a tendency to create menacing and creepy images using the psychiatric terms. For instance, the prompt incident in a psychiatric hospital showed scenes that could come from horror movies with destroyed corridors and hanging chains. On the other hand, the same prompt for a hospital showed typical scenes from emergency medicine (see Fig. 1). These outputs significantly confirm the stigma of danger and aggression in people suffering from mental health disorders.

Overall, our findings support previous evidence of bias in output of AI image generators but extend this to mental health. Since AI models extract caption-image pairings from pre-existing material in order to create new images, our results point to the stigma of mental health in the media. Possible measures to avoid the bias such as expanding the training data set or adjusting and concretising the prompts lead to mixed results [4]. In addition, the image of an ECT session in our study reveals another issue of misrepresentation—the generating of unrealistic or “too woke” images. In a most recent example, the Gemini chat bot by Google intended to create non-biased but very inaccurate images such as an Asian woman as a German soldier during World War II. Furthermore, OpenAI and Designer refused to create some of the images to avoid contributing to social biases, raising the question, if these restricting guidelines reduce or increase stigma. In the end, we need to be aware that the vetting processes of the database and the algorithms underlying these generators are still not transparent. Thus, we suggest implementation of ethical standards by the companies hosting the generators for creating images for mental health such as publishing the methods to shape model’s behaviour and companies’ policies to create AI content. Further systematic research is essential to further conceptualize these issues, to reduce stigma in AI models and adjust ethical guidelines and recommendations for users and developers.
